# Prognostic roles of pathology markers immunoexpression and clinical parameters in Hepatoblastoma

**DOI:** 10.1186/s12929-017-0369-1

**Published:** 2017-08-29

**Authors:** Jia-Feng Wu, Hsiu-Hao Chang, Meng-Yao Lu, Shiann-Tarng Jou, Kai-Chi Chang, Yen-Hsuan Ni, Mei-Hwei Chang

**Affiliations:** 1 Department of Pediatrics, National Taiwan University Hospital, No. 8, Chung-Shan S. Rd, Taipei, Taiwan; 2 Department of Emergency, National Taiwan University Hospital, Taipei, Taiwan; 3 Hepatitis Research Center, National Taiwan University Hospital, No. 8, Chung-Shan S. Rd, Taipei, Taiwan

**Keywords:** Hepatoblastoma, Liver stem cell, Epithelial cell adhesion molecule, β-catenin

## Abstract

**Background:**

Hepatoblastoma, a leading primary hepatic malignant tumor in children, is originated from primitive hepatic stem cells. We aimed to elucidate the relationships between the histological distribution of β-catenin and hepatic stem cell markers with the clinical outcomes of hepatoblastoma.

**Methods:**

Immunohistochemistry was applied to detect β-catenin and hepatic stem cell markers expression in 31 hepatoblastoma tumors. We analyzed the relationship between the stem cell markers and the clinical course of hepatoblastoma.

**Results:**

Thirty-one hepatoblastoma patients were diagnosed at a mean age of 2.58 ± 3.78 years, and 7 (22.58%) died. A lack of anticipated decrease in alpha-fetal protein levels after neoadjuvant chemotherapy indicated a higher mortality rate. Nuclear β-catenin expression was significantly associated with membranous epithelial cell adhesion molecule (EpCAM) expression in hepatoblastoma tumor specimens. The co-expression of nuclear β-catenin and membranous EpCAM together with an age at diagnosis ≤1.25 years were predictive of an alpha-fetoprotein level < 1200 ng/mL after neoadjuvant chemotherapy (*P* < 0.05). An alpha-fetoprotein level < 1200 ng/mL after neoadjuvant chemotherapy and age at hepatoblastoma diagnosis ≤1.25 years are both predictors of better overall and native liver survival in hepatoblastoma patients.

**Conclusions:**

Presence of membranous EpCAM with nuclear β-catenin and younger diagnostic age of hepatoblastoma are predictive of serum alpha-fetoprotein levels drop after chemotherapy. Younger diagnostic age and lower alpha-fetoprotein levels after neoadjuvant chemotherapy and are predictive of better overall and native liver survival in hepatoblastoma patients.

**Electronic supplementary material:**

The online version of this article (10.1186/s12929-017-0369-1) contains supplementary material, which is available to authorized users.

## Background

Hepatoblastoma is a leading hepatic malignant tumor in young children, and is believed to originate from primitive hepatic stem cells during embryogenesis of the liver. The exact pathogenesis and associated genetic factors of hepatoblastoma remain largely unknown. Although most hepatoblastoma occur sporadically, others arise in combination with Beckwith–Wiedemann syndrome or familial adenomatous polyposis [1–5].

During liver development from the fetus to newborn, the population of liver stem cells decreases gradually under fine-tuned control [1, 6]. Deregulation of this developmental process may contribute to the malignant transformation of these hepatic stem cells and result in hepatoblastoma in young children.^1^ Epithelial cell adhesion molecule (EpCAM) is both a hepatic stem cell marker and a cancer stem cell marker [1]. Upregulation of the Wnt/β-catenin pathway has been identified in 77–85% of hepatoblastoma cases according to previous study that compared tumor and non-tumor parts of the liver [1]. A difference in the cellular distribution of β-catenin has also been reported in hepatic malignancies depending on the differentiation status [3]. The deregulation of stem cell regulation genes was also demonstrated to associate with poor differentiation of liver tumor and poor clinical outcomes [7, 8].

A previous study in Taiwan demonstrated that an improved chemotherapy protocol enhances the survival rate of hepatoblastoma; however, the 2-year survival rate is still unsatisfactory [9]. A mutation in exon 3 of the β-catenin gene is associated with nuclear expression of β-catenin in sporadic hepatoblastoma patients [10]. Abnormal cellular localization of β-catenin has been reported in cholangiocarcinoma during different differentiation stages and clinical outcomes [3].

Liver cancer stem cell, have been reported to induce chemoresistance, metastasis, tumorigenesis, invasion, and poor clinical outcome [11]. OV6-positive cancer cells in human hepatocellular carcinoma (HCC) were reported to exhibit more invasive and metastatic potential, and CK19-positive cancer indicates poor surgical outcomes in patients with liver cancer [12–14]. Alter of the Wnt/β-catenin pathway has also been reported to activate the stem cell properties, cell proliferation and tumorigenesis of hepatocytes [1]. However, few reports have evaluated the relationships of the cellular localization of β-catenin and hepatic stem markers with clinical outcomes in patients with hepatoblastoma. In this study we will be assessing the pathology of liver stem cell marker immunoexpression and clinical parameters.

## Methods

### Study subjects

From 1988 to 2011, 50 children with hepatoblastoma underwent medical therapy at the Department of Pediatrics, National Taiwan University Hospital (NTUH). All patients were diagnosed by clinical and laboratory examinations [including serum alpha-fetoprotein (AFP) levels, radiography, and pathology]. The staging system at diagnosis was based on the Childhood Liver Tumor Strategy Group of the International Society of Pediatric Oncology (SIOPEL) pre-treatment extent (PRETEXT) of disease grading system [10, 15]. Tumor specimens from 31 subjects (62%) before chemotherapy were available for this study. All 31 subjects received the SIOPEL neoadjuvant chemotherapy, surgical excision (*n* = 28) /liver transplantation (*n* = 3), and SIOPEL adjuvant chemotherapy designed by the SIOPEL Group [15, 16]. The clinical outcomes and survival were assessed by a review of medical records (Table 1). The study protocol was conformed to the ethical guidelines of the 1975 Declaration of Helsinki, as reflected in a prior approval by the institution’s human research committee of NTUH. The study protocol was reviewed and approved by the Institutional Review Board of NTUH (NTUH IRB 201201023RIB).

**Table 1:** General characters of these 31 hepatoblastoma patients

	Study population (*n* = 31)
Diagnostic age, medium (IQR), years	1.0 (0.75–2.00)
Male gender, n (%)	19 (61.29%)
Histology, n (%)	
Epithelial type	23 (74.19%)
Fetal pattern	14 (45.16%)
Embryonal pattern	6 (19.35%)
Mixed fetal and embryonal pattern	3 (9.68%)
Small cell undifferentiated / anaplastic pattern	0 (0%)
Mixed Epithelial / Mesenchymal Type	8 (25.81%)
Initial maximal tumor diameter, medium (IQR), cm	9.50 (8.0–12.3)
Alpha-fetoprotein level at diagnosis, medium (IQR), log10 ng/mL	5.09 (4.53–5.76)
Maximal tumor diameter after neo-adjuvant chemotherapy, medium (IQR), cm	5 (4.0–8.2)
Alpha-fetoprotein level after neo-adjuvant chemotherapy, medium (IQR), log10 ng/mL	2.06 (1.45–3.08)
Follow up duration, medium (IQR), years	5.11 (2.97–7.16)
Mortality, n (%)	7 (22.58%)
Liver transplantation, n (%)	3 (9.68%)
Survival with native liver, n (%)	22 (70.97%)
Tumor recurrence after tumor resection, n (%)	8 (25.81%)
PRETEXT stage, n (%)	
PRETEXT I	1 (3.22%)
PRETEXT II	13 (41.94%)
PRETEXT III	10 (32.26%)
PRETEXT IV	7 (22.58%)

IQR, interquartile range; PRETEXT, PRETreatment EXTent of disease

### Pathology and Immunohistochemical staining

We assessed the expression of β-catenin and hepatic stem cell markers [(EpCAM, OV6, and cytokeratin-19 (CK19)] by immunohistochemical staining in 31 (62%) available paraffin sections of prechemotherapy hepatoblastoma tumor tissues. Tissue sections were deparaffinized and rehydrated at 100 °C using Trilogy (Cell Marque Corporation, Rocklin, CA, USA) for 10 min at 750 W. Staining was performed using rabbit-anti-EpCAM (1:50; Epitomics, Inc., Burlingame, CA, USA), mouse anti-β-catenin (1:50; Santa Cruz Biotechnology, Santa Cruz, CA, USA), mouse anti-CK19 (1:50; Novocastra Laboratories, Newcastle, UK) or mouse anti-OV6 (1:50; R&D Systems, Minneapolis, MN, USA) antibodies. The UltraVision Quanto HRP DAB Advanced Polymer Detection Kit (Thermo Fisher Scientific, Fremont, CA, USA) was used as a detection system according to the manufacturer’s protocol. Nuclei were lightly counterstained with Mayer’s hematoxylin (Biogenex, Fremont, CA, USA).

The pattern of β-catenin expression was classified into three categories: (1) preserved membranous expression pattern, (2) reduced membranous expression pattern, or (3) nuclear expression pattern [4]. For EpCAM, CK19, and OV6, positive staining was defined as membranous and/or cytoplasmic staining in ≥5% of tumor cells of moderate or strong intensity [12, 13, 17]. We further assessed the impact of the histological subtype, β-catenin pattern, and hepatic stem cell markers (EpCAM, OV6, and CK19) on clinical outcomes.

### Dual staining of β-catenin and EpCAM by immunofluorescence

Tissue sections were deparaffinized and rehydrated at 100 °C using Trilogy (Cell Marque Corporation) for 10 min at 750 W. Sections were then blocked with 5% bovine serum albumin for 30 min at room temperature. Staining was performed using rabbit-anti EpCAM (1:50; Epitomics, Inc.) and mouse anti-β-catenin (1:50; Santa Cruz Biotechnology) antibodies. We also labeled nucleic acid by 4′,6-diamidino-2-phenylindole (DAPI). After washing with phosphate-buffered saline, slides were incubated with Alexa Fluor 488- or 594-conjugated secondary antibodies (1:200; Invitrogen, Carlsbad, CA, USA) for 1.5 h. Slides were then washed and mounted. The microscope (Axio Observer D1, Carl Zeiss, Berlin, Germany) was applied to read the image.

### Statistical analysis

STATA (version 14, StataCorp LP, College Station, TX, USA) and MedCalc (version 17.5.0; MedCalc Software, Ostend, Belgium) software were used for the statistical analyses. Student’s t-test with unequal variance was used to analyze differences in the means and 95% confidence intervals (95% CIs) or Mann-Whitney U test for the differences in median/interquartile range (IQR) of continuous data, and Fisher’s exact test was used to analyze differences in the rates of categorical variables. Receiver operating characteristic (ROC) curve analysis was used to determine cutoff levels. Native liver survival was defined as the survival of patients with their native liver, while overall survival was defined as the survival of patients regardless of whether liver transplantation was performed. Survival analysis for native liver survival and overall survival were analyzed by Cox’s proportional hazard method and Kaplan-Meier plots. Bonferroni correction was applied to adjust the significant *P* values in the statistical models involving multiple comparisons.

## Results

### Clinical findings

The clinical findings in thirty-one subjects with available pre-chemotherapy tumor specimens are summarized in Table 1. All study subjects included in this study had high initial serum AFP levels at the diagnosis of hepatoblastoma (Table 1). The tumor size of the study population decreased significantly after SIOPEL neoadjuvant chemotherapy (6.03 ± 2.69 vs. 10.02 ± 3.47 cm in diameter; *P* < 0.001). The serum AFP levels also decreased significantly after neoadjuvant chemotherapy (4.95 ± 1.19 vs. 2.57 ± 1.25 ng/mL in Log10 ratio; *P* < 0.001).

All study subjects (*n* = 31) received SIOPEL neoadjuvant chemotherapy, of whom 22 (70.97%) survived with their native livers after SIOPEL neoadjuvant chemotherapy, surgical total tumor resection, and adjuvant chemotherapy. Three (9.68%) children underwent liver transplantation as a result of a non-resectable tumor after neoadjuvant chemotherapy (*n* = 1) or tumor recurrence (*n* = 2). One patient who received a liver transplant died of post-transplantation-related acute myeloblastic leukemia. Another 6 children (19.35%) died with their native livers as a result of distant metastasis, tumor rupture, or the lack of an adequate liver donor.

Native liver survivors (*n* = 22) had a younger age at diagnosis than did those without native liver survival (*n* = 9) (Table 2, *P* = 0.004). Native liver survivors also had lower serum AFP levels after neoadjuvant chemotherapy than did those without native liver survival (*P* = 0.04). There was no obvious difference between native liver survivors and those without native liver survival in terms of sex, initial tumor size, initial AFP levels, maximum tumor size after neoadjuvant chemotherapy, or PRETEXT stage (Table 2).

**Table 2:** Difference in clinical characters between native liver survivors and other subjects in this hepatoblastoma cohort

	Survive with Native liver (*n* = 22)	Others (*n* = 9)	*p*-value
Diagnostic age, medium (range), years	1 (0–10.0)	2 (1–15.0)	0.004
Male gender, n (%)	15 (68.2)	4 (44.4%)	0.25
Initial maximal tumor diameter, medium (range), cm	9.50 (4.20–20.1)	9.90 (5.0–13.0)	0.78
Initial AFP level, medium (range), log10 ng/mL	5.23 (3.33–6.16)	5.09 (1.30–6.47)	0.84
Maximal tumor size after neo-adjuvant chemotherapy, medium (range), cm	5.00 (2.6–11.0)	7.40 (1.50–12.0)	0.31
AFP level after neo-adjuvant chemotherapy, medium (range), log10 ng/mL	2.01 (1.15–4.58)	3.44 (1.18–6.55)	0.04
PRETEXT stage*, n (%)			
PRETEXT I	1 (4.55%)	0 (0%)	
PRETEXT II	10 (45.45%)	3 (33.33%)	
PRETEXT III	7 (31.82%)	3 (33.33%)	
PRETEXT IV	4 (18.18%)	3 (33.33%)	0.73

*PRETEXT, PRETreatment EXTent of disease

A greater reduction in serum AFP levels after neoadjuvant chemotherapy was correlated with smaller tumor size after neoadjuvant chemotherapy (correlation coefficient = 0.41; *P* = 0.02). Native liver survivors exhibited a greater decrease in AFP levels than did the subjects without native liver survival (*P* = 0.02).

### Immunohistochemical staining

**Fig. 1:**
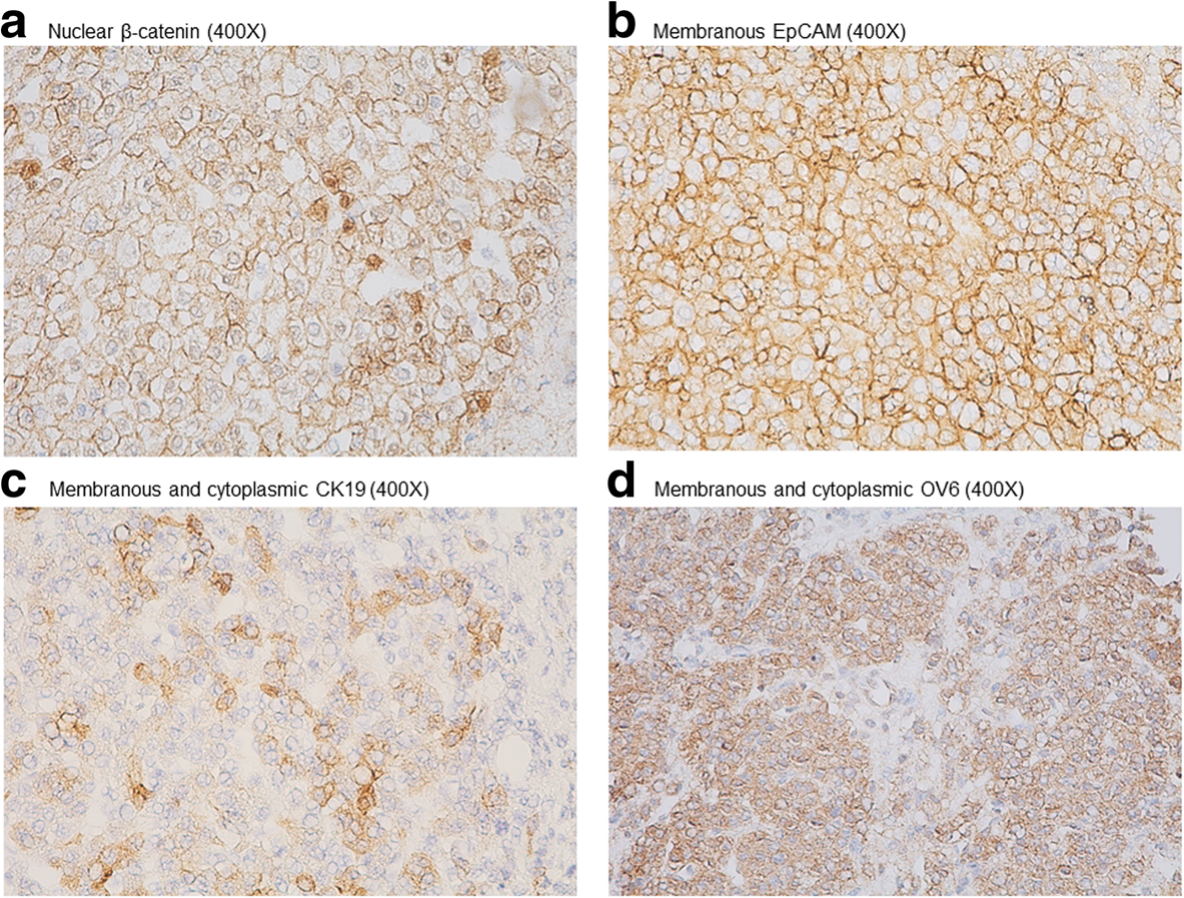
Immunohistochemical staining. **a** Nuclear staining of β-catenin. **b** Membranous staining of EpCAM. **c** Membranous and cytoplasmic staining of CK19. **d** Membranous and cytoplasmic staining of OV6

**Fig. 2:**
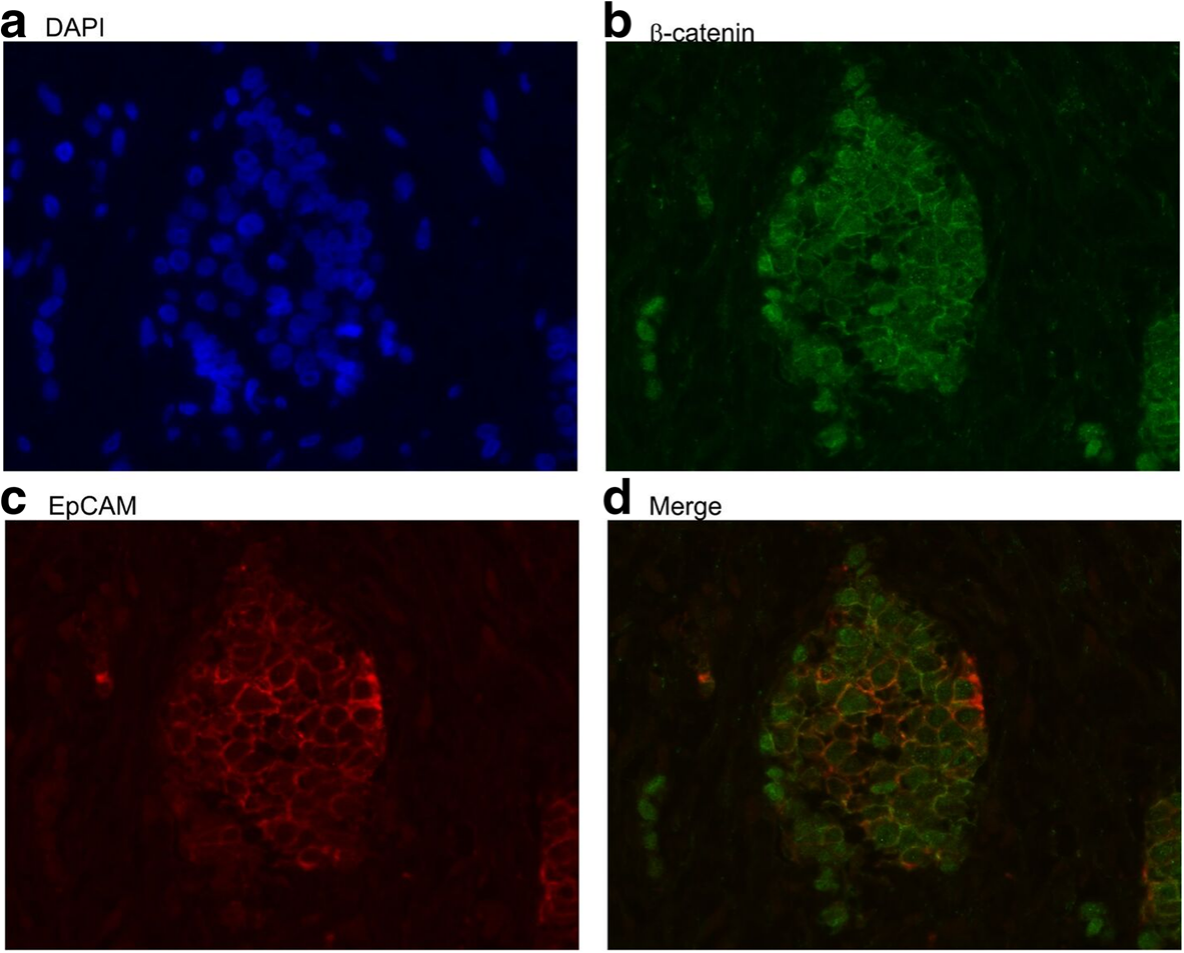
Immunohistochemical staining was applied to showe the relationship between the location of β-catenin and EpCAM. **a** Labeling nucleic acid by 4′,6-diamidino-2-phenylindole (DAPI) **b** Positive nuclear staining of β-catenin. **c** Positive membranous staining of EpCAM. **d** Dual positive nuclear staining of β-catenin and membranous staining of EpCAM

Immunohistochemical staining of 31 prechemotherapy tumor specimens showed positive membranous EpCAM staining in 25 (80.65%) specimens and negative in 6 (19.35%). The β-catenin was detected in both the nucleus and cytoplasm in 24 (77.42%) subjects. CK19 staining was positive in 25 (80.65%) specimens and negative in 6 (19.35%). OV6 staining was positive in 27 (87.10%) specimens and negative in 4 (12.90%). There was no obvious difference in the expression patterns of these markers between patients with the pure epithelial type and those with the mixed epithelial/mesenchymal type hepatoblastoma (*P* > 0.05). The positive staining patterns of nuclear β-catenin, membranous EpCAM, and membranous and/or cytoplasmic CK19 and OV6 are shown in Fig. 1. The dual positive patterns of immunofluorescence staining of membranous EpCAM and nuclear β-catenin are shown in Fig. 2. The expression pattern of stem cell markers in these tumor specimens were summarized in Additional file 1: Table S1.

Membranous EpCAM staining was positively correlated with nuclear β-catenin localization in our study population (correlation coefficient = 0.52; *P* = 0.003), and nuclear β-catenin localization was also predictive of the presence of membranous EpCAM (Odds ratio, 14.67; 95% CI: 2.07–104.86; *P* = 0.01) in hepatoblastoma tumor specimens. No significant relationship between positive OV6 and CK19 staining and nuclear β-catenin staining was noted in these study subjects (*P* > 0.05).

### Clinical outcomes

**Fig. 3:**
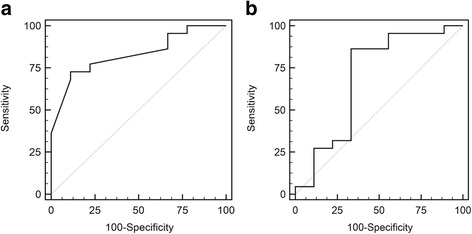
**a** Receiver operating characteristic (ROC) analysis yielded a cutoff age at diagnosis ≤1.25 years, which achieved the best prediction of native liver survival (sensitivity, 72.7%; specificity, 88.9%; area under the curve, 83.0%; *P* < 0.001). **b** ROC analysis yielded a cutoff alpha-fetoprotein level of <1200 ng/mL, which achieved the best prediction of native liver survival (sensitivity, 86.4%; specificity, 66.7%; area under the curve, 69.2%; *P* = 0.04)

The ROC analysis also yielded a cutoff age at diagnosis ≤1.25 years, which achieved the best prediction of survival of patients with their native liver (sensitivity, 72.7%; specificity, 88.9%; area under the curve, 83.0%, *P* < 0.001, Fig. 3a). ROC analysis yielded a cutoff AFP level of <1200 ng/mL after SIOPEL neoadjuvant chemotherapy, which achieved the best prediction of survival of patients with their native liver (sensitivity, 86.4%; specificity, 66.7%; area under the curve, 69.2%, *P* = 0.04, Fig. 3b).

Subjects with positive staining of both membranous EpCAM and nuclear β-catenin were more likely to have an AFP level < 1200 ng/mL after neoadjuvant chemotherapy (Odds ratio, 9; 95% CI: 1.66–49.12; *P* = 0.01). Diagnosis of hepatoblastoma at ≤1.25 years of age was also associated with an AFP level < 1200 ng/mL after neoadjuvant chemotherapy (Odds ratio, 8; 95% CI: 1.33–48.18; *P* = 0.02) in this study.

**Fig. 4:**
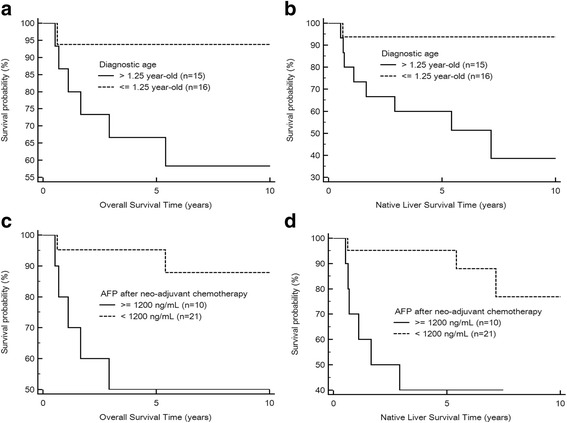
**a** A Kaplan-Meier plot demonstrates that the overall survival rate was significantly higher in hepatoblastoma patients with an age at diagnosis ≦ 1.25 years compared with the those with diagnostic age > 1.25 years (*P* = 0.03). **b** The native liver survival rate was significantly higher in patients with hepatoblastoma and an age at diagnosis ≦ 1.25 years compared with the those with diagnostic age > 1.25 years (*P* = 0.007). **c** The overall survival rate was significantly higher in patients with hepatoblastoma and serum alpha-fetoprotein levels <1200 ng/mL compared with those with serum alpha-fetoprotein levels ≧ 1200 ng/mL after neuadjuvant chemotherapy (*P* = 0.01). **d** The native liver survival rate was significantly higher in patients with hepatoblastoma and serum alpha-fetoprotein levels <1200 ng/mL compared with those with serum alpha-fetoprotein levels≧ 1200 ng/mL after neuadjuvant chemotherapy (*P* = 0.004)

An age at diagnosis of hepatoblastoma ≤1.25 years was also associated with overall survival (*P* = 0.03 by log-rank test; Fig. 4a) and native liver survival (*P* = 0.007 by log rank test; Fig. 4b). Kaplan-Meier analysis indicated the predictive role of an AFP level < 1200 ng/mL after SIOPEL neoadjuvant chemotherapy on overall survival (*P* = 0.01 by log-rank test; Fig. 4c) and native liver survival (*P* = 0.004 by log-rank test; Fig. 4d). The 5- and 10-year overall survival rates were 95% and 85%, respectively, of hepatoblastoma subjects with an AFP level < 1200 ng/mL after SIOPEL neoadjuvant chemotherapy. While the 5- and 10-year overall survival rates were 50% and 50%, respectively, of hepatoblastoma subjects with an AFP level ≧ 1200 ng/mL after SIOPEL neoadjuvant chemotherapy.

The 5- and 10-year native liver survival rates were 95% and 75%, respectively, of hepatoblastoma subjects with an AFP level < 1200 ng/mL after SIOPEL neoadjuvant chemotherapy. While the 5- and 10-year native liver survival rates were 40% and 40%, respectively, of hepatoblastoma subjects with an AFP level ≧ 1200 ng/mL after SIOPEL neoadjuvant chemotherapy.

Cox’s proportional hazard analysis further confirmed these results (Table 3). A serum AFP level < 1200 ng/mL after SIOPEL neoadjuvant chemotherapy and no tumor recurrence were demonstrated to be predictors of native liver survival (hazard ratio: 4.54 and 5.55; *P* = 0.04 and 0.02; respectively) on Cox’s proportional hazard analysis (Table 3).

**Table 3:** The predictors of overall survival and native liver survival time in hepatoblastoma children receiving SIOPEL chemotherapy

Native liver survival	Univariate analysis*			Multivariate analysis		
	Hazard ratio	95% CI	*p*-value	Hazard ratio	95% CI	*p*-value
AFP levels <1200 ng/mL (*n* = 21) vs. ≧ 1200 ng/mL (*n* = 10) after SIOPEL neo-adjuvant chemotherapy	6.01	1.49–24.28	0.01	4.54	1.05–19.64	0.04
Female (*n* = 12) vs. male (*n* = 19)	0.36	0.10–1.39	0.14	-	-	-
Age at diagnosis <=1.25 year-old (*n* = 16) vs. > 1.25 year-old (*n* = 15)	10.06	1.26–80.54	0.03	-	-	-
No tumor recurrence (*n* = 23) vs. Tumor recurrence (*n* = 8) after tumor resection	7.23	1.80–29.06	0.005	5.55	1.32–23.29	0.02
Overall survival	Univariate Analysis			Multivariate Analysis		
	Hazard ratio	95% CI	*p*-value	Hazard ratio	95% CI	*p*-value
AFP levels <1200 ng/mL (*n* = 21) vs. ≧ 1200 ng/mL (*n* = 10) after SIOPEL neo-adjuvant chemotherapy	6.59	1.27–34.11	0.02	-	-	-
Female (*n* = 12) vs. male (*n* = 19)	0.18	0.03–0.97	0.04	-	-	-
Age at diagnosis <=1.25 year-old (*n* = 16) vs. > 1.25 year-old (*n* = 15)	7.00	0.84–58.19	0.07	-	-	-
No tumor recurrence (*n* = 23) vs. Tumor recurrence (*n* = 8) after tumor resection	4.22	0.94–18.94	0.06	-	-	-

*Bonferroni correction was applied to adjust the *P* value in multiple comparison in univariate analysis of this analysis. The *P* value was adjusted to <0.0125 as statistical significant and 0.0125–0.025 as borderline significance in univariate analysis, and only variable with significant *P* value were forwarded to the multivariate analysis

## Discussion

Here we demonstrate that an age at diagnosis ≤1.25 years and serum AFP levels <1200 ng/mL after SIOPEL neoadjuvant chemotherapy are both predictors of better overall and native liver survival of patients with hepatoblastoma with high initial serum AFP levels. The expression levels of β-catenin, EpCAM, CK19, and OV6 in our hepatoblastoma specimens are high, indicating that hepatoblasts in the early phase of liver development are a possible origin of hepatoblastoma cancer stem cells. Double positive staining of nuclear β-catenin and membranous EpCAM was associated with an AFP level < 1200 ng/mL after SIOPEL neoadjuvant chemotherapy.

Activation of the Wnt/β-catenin pathway is reportedly associated with liver carcinogenesis [7, 9, 17–20]. Altered cellular distribution of β-catenin has been reported in hepatic malignancies at different differentiation stages [6, 20]. Recent whole-exome sequencing studies showed a high prevalence of β-catenin(*CTNNB1*) gene mutations in hepatoblastoma tumor specimens [18, 19]. Up to 89% of hepatoblastoma tumors in Taiwan have been reported to contain mutations, including deletions and missense mutations, in exon 3 of the β-catenin (*CTNNB1*) gene, and 87% in a Western study were reported to carry mutations within the ubiquitination domain of the β-catenin (*CTNNB1*) gene [10, 20]. These data indicate the important roles of β-catenin in the tumorigenesis of hepatoblastoma. A recent immunohistochemistry study also showed that up to 83.6% of hepatoblastoma tumor specimens stained positive for EpCAM, a rate similar to that in our study [17]. EpCAM-positive cancer cells have been demonstrated to associate with increase self-renewal and tumorigenesis capacities [11, 21]. Recent studies showed that EpCAM expression may be regulated by the Wnt/β-catenin signaling pathway in hepatoma cells [22, 23]. Gene expression profiling and pathway analysis showed that EpCAM-positive HCC displays a distinct molecular signature with features associated with hepatic stem cells, including the expression of known stem cell markers and activation of Wnt/β-catenin signaling [22]. In our study population, we showed that the presence of membranous EpCAM is highly correlated with the expression of nuclear β-catenin in hepatoblastoma. A previous study demonstrated that nuclear accumulation of β-catenin may activate the expression of EpCAM in liver cancer cells, which is consistent with our findings [22]. Co-expression of membranous EpCAM and nuclear β-catenin in the tumor specimens in our study population was associated with an AFP level < 1200 ng/mL after SIOPEL neoadjuvant chemotherapy, indicating improved chemosensitivity.

A diagnosis of hepatoblastoma at ≤1.25 years of age was also associated with an AFP level < 1200 ng/mL after SIOPEL neoadjuvant chemotherapy (Odds ratio: 8) in this study. Hence, subjects with earlier hepatoblastoma onset (≤1.25 years) may have greater chemosensitivity, a greater likelihood of AFP levels <1200 ng/mL after neoadjuvant chemotherapy, and thus improved native liver and overall survivals as compared with those diagnosed after 1.25 years.

A low initial serum AFP level (<100 ng/mL) at the diagnosis of hepatoblastoma was reported to be a poor prognostic indicator [24]. There are also several new poor prognostic markers in hepatoblastoma reported by Children’s Hepatic tumors International Collaboration (CHIC) in recent years, including diagnostic age (≧8 years in PRETEXT I-III, and ≧ 3 years in PRETEXT IV), initial AFP level (≤1000 ng/mL in PRETEXT I-III, and ≤100 ng/mL in PRETEXT IV) [25–27]. In our study, there are 24 subjects graded as PRETEXT I-III, and only 2 (9.32%) of them were diagnosed at the age ≧ 8 years. There is no significant difference between PRETEXT I-III subjects with diagnostic age < 8 vs. ≧ 8 years (*P* = 0.054 and 0.054 for overall survival and native liver survival, respectively). There are 7 subjects graded as PRETEXT IV, and only 2 (28.57%) of them were diagnosed at the age ≧ 3 years. The overall and native liver survival rate is also insignificantly different between PRETEXT IV subjects <3 vs. ≧ 3 years (*P* = 1.000 and 1.000, respectively. All subjects in our group had a high initial AFP level at diagnosis (>1000 ng/mL). Hence, the cutoff diagnostic age and cutoff initial AFP reported recently did not act as feasible prognostic indicators in our hepatoblastoma population in Taiwan [25–27]. Those subjects with serum AFP level decreased to <1200 ng/mL after SIOPEL neoadjuvant chemotherapy was associated with high 5- and 10-year overall and native liver survival rates in the population with high initial serum AFP levels compared with others with serum AFP levels ≧ 1200 ng/mL after SIOPEL neoadjuvant chemotherapy in this study and in previous reports under various chemotherapy regimens [28–32]. This implies that hepatoblastoma of different differentiation stages may have different responses to chemotherapy. Patients with an earlier age of hepatoblastoma onset (≤1.25 years) who are double positive for membranous EpCAM and nuclear β-catenin in tumors have a better response to chemotherapy and better long-term outcomes.

We failed to demonstrate a relationship between cell markers and tumor size after neoadjuvant chemotherapy, which is most likely due to the differentiation of hepatoblastoma cells into mature hepatocytes and calcification/fibrosis of the tumor mass. Hence, the tumor size does not reflect tumor burden after neoadjuvant chemotherapy in hepatoblastoma.

The limitation of this study is its relatively small sample size. We did not have adequate statistical power to demonstrate significant relationships, either by Cox’s proportional hazard survival or logistic regression analysis, of the hepatoblastoma histology subtype and β-catenin, EpCAM, OV6, and CK19 expression with overall survival and native liver survival rates. However, we did identify a relationship between the expression pattern of different cell markers and chemosensitivity in patients with hepatoblastoma. A large-scale study may be needed to clarify the direct relationships among histology subtype, cell markers, and the survival of patients with hepatoblastoma.

## Conclusions

We identified a subgroup of hepatoblastoma patients with early-onset age (≤1.25 years) and double positive expression of membranous EpCAM and nuclear β-catenin in tumors with better chemosensitivity and better overall and native liver survival rates. Hepatoblastoma subjects with a high initial serum AFP level and decreases to <1200 ng/mL after neoadjuvant chemotherapy is a good clinical prognostic marker.

## Additional file

Additional file 1: Table S1. The expression of stem cell markers in the tumor specimens from these 31 subjects. (DOC 46 kb)

## Abbreviations

AFP: Alpha-fetoprotein
EpCAM: Epithelial cell adhesion molecule
HCC: human hepatocellular carcinoma
