# Supplementary material for: Prognostic roles of pathology markers immunoexpression and clinical parameters in Hepatoblastoma
Source: J Biomed Sci. 2017 Aug 29;24:62. doi: 10.1186/s12929-017-0369-1 (PMC5574230; doi:10.1186/s12929-017-0369-1)
Supplement: Additional file 1: Table S1. — The expression of stem cell markers in the tumor specimens from these 31 subjects. (DOC 46 kb) [file 12929_2017_369_MOESM1_ESM.doc]

| Subject number | Nuclear β-catenin | Membranous EpCAM | OV6 | CK19 |
| --- | --- | --- | --- | --- |
| 1 | + | + | + | + |
| 2 | + | + | + | + |
| 3 | + | + | + | + |
| 4 | + | + | + | + |
| 5 | + | + | + | + |
| 6 | + | + | + | + |
| 7 | + | + | + | + |
| 8 | + | + | + | + |
| 9 | + | + | + | + |
| 10 | + | + | + | + |
| 11 | + | + | + | + |
| 12 | + | + | + | + |
| 13 | + | + | + | + |
| 14 | + | + | + | - |
| 15 | + | + | + | - |
| 16 | + | + | + | - |
| 17 | + | + | + | - |
| 18 | + | + | - | + |
| 19 | + | + | - | + |
| 20 | + | + | - | + |
| 21 | + | + | + | + |
| 22 | + | + | + | + |
| 23 | + | - | + | + |
| 24 | + | - | + | + |
| 25 | - | + | + | + |
| 26 | - | + | + | + |
| 27 | - | + | - | - |
| 28 | - | - | + | + |
| 29 | - | - | + | + |
| 30 | - | - | + | + |
| 31 | - | - | + | - |
